# Microscope integrated OCT (Mi-OCT) guided retrieval of intrastromal corneal foreign body

**DOI:** 10.3205/oc000217

**Published:** 2023-03-01

**Authors:** Ashish Markan, Kiran Chandra, Ramandeep Singh, Mohit Dogra

**Affiliations:** 1 Advanced Eye Centre, Post Graduate Institute of Medical Education and Research, Chandigarh, India

**Keywords:** Mi-OCT, sugarcane foreign body, intrastromal foreign body

## Abstract

**Introduction:**

To describe the role of microscope integrated optical tomography (Mi-OCT) in removal of intrastromal corneal foreign body.

**Methodology:**

A young male presented with trauma to the right eye with sugarcane stick. Ocular examination revealed two sugarcane particles, approximately 3.5 mm in greatest dimension, embedded in the corneal stroma. For removal of foreign body, Mi-OCT was switched on and the area of interest was focused. Sugarcane particles appeared as hyperreflective structures embedded in the corneal stroma with everted edges of the overlying corneal epithelium and anterior stroma.

**Results:**

Both the sugarcane particles were removed successfully under real time images provided by Mi-OCT without causing any inadvertent damage to the corneal stroma.

**Conclusion:**

Mi-OCT can be used as an adjunct in emergency surgical procedures like removal of intrastromal corneal foreign bodies with accurate precision.

## Introduction

Mi-OCT has emerged as a useful tool in providing surgeons with real-time cross sectional images of ocular tissues intraoperatively [1], [2], [3]. It has been successfully used in both anterior and posterior segment surgeries and helps surgeons to take important intraoperative decisions, thus minimizing both intraoperative and postoperative complications [4], [5]. We describe a case where Mi-OCT was used to aid us in the retrieval of an organic intrastromal corneal foreign body (FB).

## Case description

**Figure 1:**
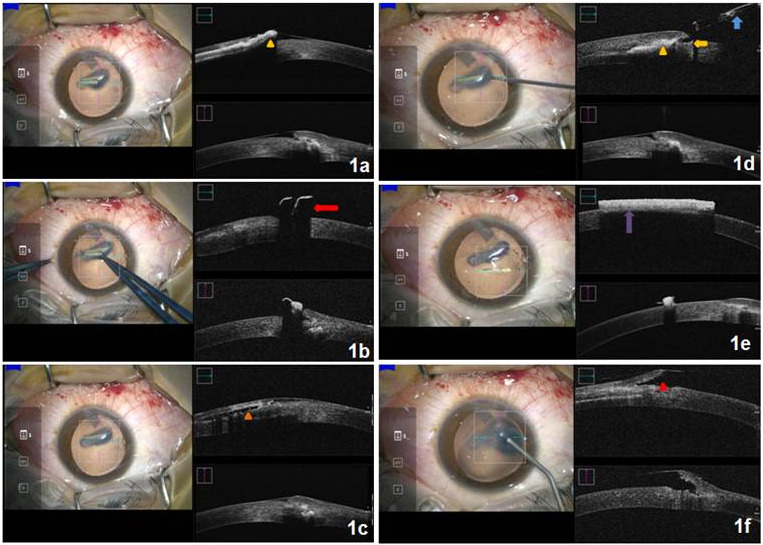
1a shows a hyperreflective structure (yellow arrow head) embedded in the anterior cornea, suggestive of an intracorneal sugarcane particle. The proximal end of the particle can be seen piercing through the corneal epithelium and anterior stroma causing a lamellar laceration. 1b shows that the sugarcane particle was held using Lim’s forceps (red arrow) while looking at the real-time images provided by Mi-OCT. 1c shows the tract of the sugarcane particle which appeared as a hyporeflective space (orange arrowhead) in the anterior cornea. 1d shows the second sugarcane particle which appeared as a hyperreflective structure (yellow arrowhead), with a narrow tract (yellow arrow). ILM forceps (blue arrow) was guided using the real-time images to retrieve it atraumatically. 1e shows the sugarcane particle lying on the corneal surface after removal. It appeared hyperreflective with back shadowing. 1f shows lamellar tissue defect (red arrowhead) once both foreign bodies were removed.

A 32-year-old male presented with a history of trauma to the right eye (OD) with a sugarcane stick 1 day back. His best corrected visual acuity in OD was 6/18 and in the left eye (OS) was 6/6. By non-contact tonometry, intraocular pressure in OD and OS were 14 and 16 mmHg, respectively. Slit-lamp examination revealed two sugarcane particles, approximately 3.5 mm in greatest dimension, embedded in the corneal stroma. These were lying horizontally in the paracentral cornea, just inferior to the visual axis. The tract through which these sugarcane particles entered the cornea was, however, not evident on slit-lamp examination. The patient was counseled, and after obtaining written informed consent, he was taken up for removal of the intrastromal corneal sugarcane particles under the operating microscope. Mi-OCT was switched on and the area of interest was focused. Sugarcane particles appeared as hyperreflective structures embedded in the corneal stroma with everted edges of the overlying corneal epithelium and anterior stroma (Figure 1a (Fig. 1)). The first FB was removed using Lim’s forceps under real-time Mi-OCT image guidance (Figure 1b (Fig. 1)), thus preventing inadvertent damage to the surrounding corneal tissue. Once the FB was removed, its tract could be clearly appreciated on the Mi-OCT as a hyporeflective lucency in the anterior cornea (Figure 1c (Fig. 1)). Subsequently the second organic FB was imaged with Mi-OCT and a decision to remove it with 25 gauge internal limiting membrane (ILM) forceps was made since the tract through which it entered was very narrow (Figure 1d (Fig. 1)). ILM forceps were used to hold the sugarcane particle and to remove it “in-toto” without causing any breakage or iatrogenic trauma to the surrounding tissues. The lamellar epithelial and anterior stromal tissue defect (Figure 1f (Fig. 1)) was irrigated with 0.5% preservative-free moxifloxacin and balanced salt solution, and a 14 mm bandage contact lens was placed.

## Conclusion

This report highlights the use of Mi-OCT in the retrieval of two organic intrastromal corneal FBs in a patient where the entry tract could not be appreciated on pre-operative examination. Attempts to remove such organic FBs on the slit-lamp can cause collateral damage to the surrounding corneal tissue and compromise visual outcomes. Mi-OCT can help in the successful removal of such intrastromal corneal FBs by providing real-time visualization of corneal planes, thereby avoiding damage to the surrounding healthy tissue and aiding in relatively atraumatic removal of organic FBs.

## Notes

### Authors’ contributions

All authors have contributed equally in the preparation of the manuscript.

### Competing interests

The authors declare that they have no competing interests.
